# Memory T Cells in Pregnancy

**DOI:** 10.3389/fimmu.2019.00625

**Published:** 2019-04-02

**Authors:** Tom E. C. Kieffer, Anne Laskewitz, Sicco A. Scherjon, Marijke M. Faas, Jelmer R. Prins

**Affiliations:** ^1^Department of Obstetrics and Gynecology, University Medical Center Groningen, University of Groningen, Groningen, Netherlands; ^2^Division of Medical Biology, Department of Pathology and Medical Biology, University Medical Center Groningen, University of Groningen, Groningen, Netherlands

**Keywords:** pregnancy, reproduction, memory T cell, immunologic memory, literature review

## Abstract

Adaptations of the maternal immune response are necessary for pregnancy success. Insufficient immune adaption is associated with pregnancy pathologies such as infertility, recurrent miscarriage, fetal growth restriction, spontaneous preterm birth, and preeclampsia. The maternal immune system is continuously exposed to paternal-fetal antigens; through semen exposure from before pregnancy, through fetal cell exposure in pregnancy, and through microchimerism after pregnancy. This results in the generation of paternal-fetal antigen specific memory T cells. Memory T cells have the ability to remember previously encountered antigens to elicit a quicker, more substantial and focused immune response upon antigen reencounter. Such fetal antigen specific memory T cells could be unfavorable in pregnancy as they could potentially drive fetal rejection. However, knowledge on memory T cells in pregnancy has shown that these cells might play a favorable role in fetal-maternal tolerance rather than rejection of the fetus. In recent years, various aspects of immunologic memory in pregnancy have been elucidated and the relevance and working mechanisms of paternal-fetal antigen specific memory T cells in pregnancy have been evaluated. The data indicate that a delicate balance of memory T cells seems necessary for reproductive success and that immunologic memory in reproduction might not be harmful for pregnancy. This review provides an overview of the different memory T cell subtypes and their function in the physiology and in complications of pregnancy. Current findings in the field and possible therapeutic targets are discussed. The findings of our review raise new research questions for further studies regarding the role of memory T cells in immune-associated pregnancy complications. These studies are needed for the identification of possible targets related to memory mechanisms for studies on preventive therapies.

## Introduction

**Figure 1:**
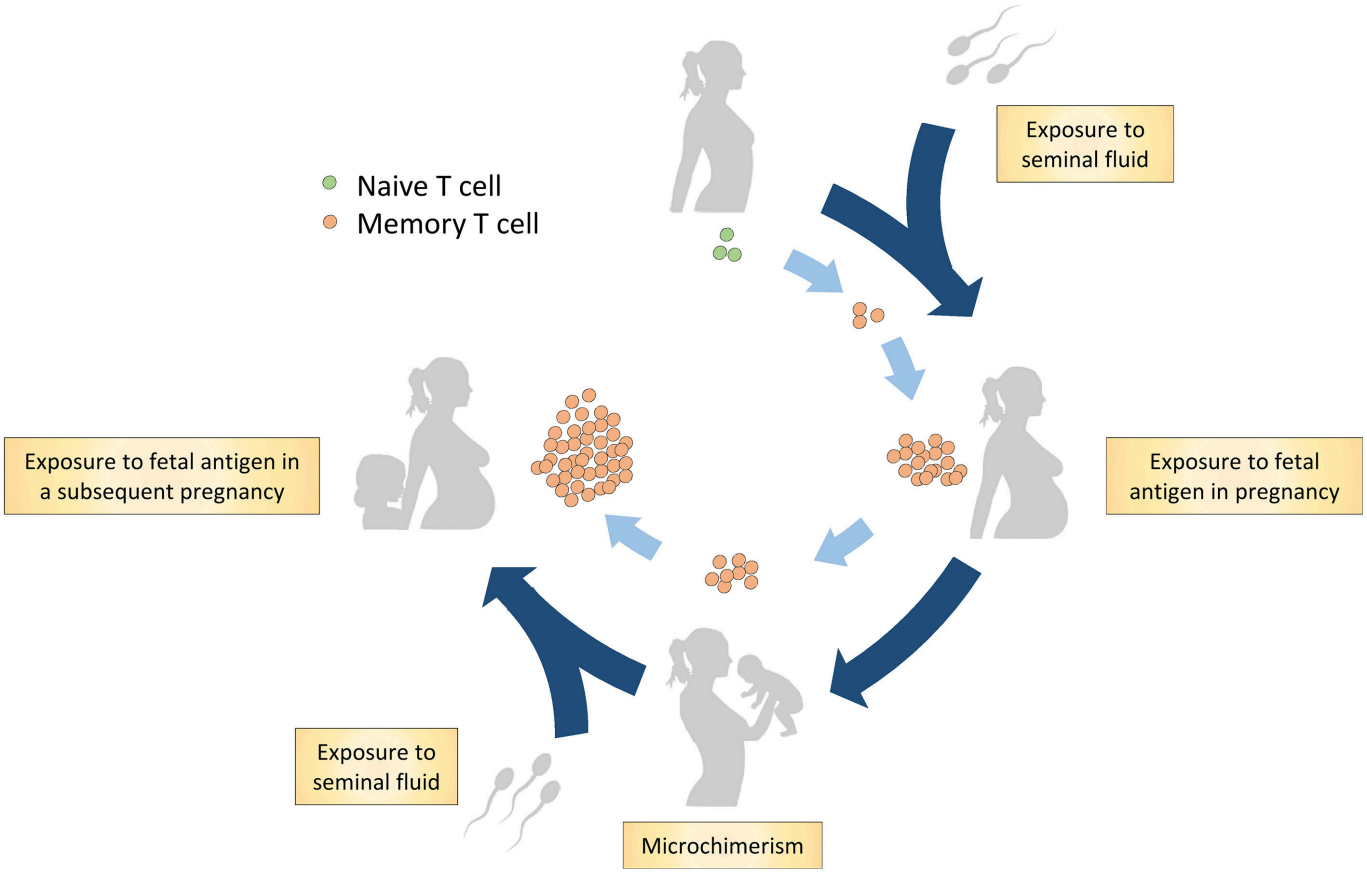
Hypothesis on generation of the memory T cell population in reproduction through paternal-fetal antigen exposure. Firstly, naive T cells are exposed to the male antigen through antigens in seminal fluid. A subsequent encounter with the antigens occurs during pregnancy through exposure to fetal antigens on trophoblast cells and through microchimerism. Postpartum, the maternal immune system remains exposed to fetal antigens through microchimerism. In addition, postpartum, memory T cells are possibly exposed to paternal antigens through exposure to seminal fluid. In a subsequent pregnancy, the maternal memory T cells likely reaccumulate and respond to the cognate paternal-fetal antigens.

Immune tolerance toward paternal-fetal antigen is crucial for reproductive success since dysfunctional tolerance is implicated in the pathophysiology of pregnancy complications as infertility, recurrent miscarriage, fetal growth restriction, spontaneous preterm birth, and preeclampsia (1–4). In reproduction, the maternal immune system is exposed to paternal-fetal antigens (Figure 1). Firstly, the male antigen is introduced to the maternal immune system through semen exposure even before pregnancy (5). Secondly, paternal-fetal antigens are exposed at the fetal-maternal interface in pregnancy since the maternal immune cells in blood are in direct contact with fetal trophoblast cells in the placenta (6, 7). Additionally, in pregnancy, there is trafficking of fetal cells expressing paternal-fetal antigens to maternal tissues at low levels which can recirculate in the maternal blood for years after pregnancy (8, 9). This phenomenon is called microchimerism (8, 9). It has been shown that the exposure of the maternal immune system to paternal-fetal antigens induces a memory T cell population with paternal-fetal antigen specificity (10–12).

The memory lymphocyte population is comprised of memory T lymphocytes (T cells) and memory B lymphocytes (B cells) (13, 14). Memory T cells are the most studied and appear to be the most important memory cell population in reproduction. Memory cells enable the immune system to protect the body from pathogens efficiently by generating a more adequate immune response to a known antigen, making it unnecessary to elicit a new response to an antigen that was encountered before (15). This process forms the basis for vaccination which is widely used to prevent infectious diseases and more recently to fight cancer and auto-immune diseases (16–18). In general, a more aggressive immune response toward pathogens is protective for health since the pathogen is cleared faster, however, the same aggressive response toward paternal- or fetal antigens would be disastrous for fetal and maternal health. Indeed, most studies of memory T cell populations in reproduction indicated that memory T cell subsets may exhibit a different function, proliferation pattern and migratory abilities toward paternal antigens in healthy pregnancies as compared with their function, proliferation and migratory abilities toward other antigens (12, 19, 20). In fact, specific memory cell populations have been shown to be involved in generating immune tolerance, rather than immune rejection, toward paternal-fetal antigens (12, 21–23).

In recent years, the implication and relevance of memory T cells in pregnancy and complications of pregnancy have been revealed. Major conceptual breakthroughs were seen in the T cell field, showing the role of memory T cells in reproductive fitness in mouse studies (11, 12, 21). Since increasing numbers of human studies on memory T cells have been published, this review gives an overview of the current literature on the different memory T cell subtypes and their adaptation in pregnancy and the implication of memory T cells in different complications of pregnancy. We will mainly focus on human studies and refer to mouse studies if needed. Current research gaps, controversies, and possible therapeutic targets will also be discussed.

## Memory T Cells

The memory T cell population is formed during a primary antigen response (24). In the primary response, antigens are presented to T cells through major histocompatibility complex (MHC) molecules (25). Depending on the type of MHC molecule, either type I or type II, CD8 positive or CD4 positive T cells respectively are activated through the T cell receptor (TCR) on the cell membrane (25). Additional co-stimulatory molecules can connect to co-stimulatory receptors on the T cell such as CD28 and CD70, for extra induction of the T cell response (25, 26). Depending on the cytokine environment, CD4^+^ cells differentiate into either different T helper (Th) subsets (Th1, Th2, and Th17) which help in inducing/activating immune responses through secretion of cytokines, or into T regulatory (Treg) cells which exert regulatory effects on other immune cells after activation (27). After the primary response, most CD4^+^ cells die, but some CD4^+^ cells differentiate into CD4^+^ memory T cells (24, 28). CD8^+^ cells also differentiate into different subpopulations; i.e., effector CD8^+^ cells which are ready to release cytotoxic cytokines or induce apoptosis via cell surface interaction, and a small population of regulatory CD8^+^ cells which exhibit an immune regulatory function (29). Once the pathogen is cleared, most CD8^+^ cells die, however some proliferate into memory CD8^+^ cells (29).

Several memory T cell subsets are known, and can be distinguished by various markers (Tables 1, 2). The main markers are CD45RO expression, and lack of CD45RA expression (52, 53). The CD45RO^+^CD45RA^−^ phenotype has been linked to long living memory T cells (52, 53). It should be noted that CD45RO expression and lack of CD45RA expression are not conclusive markers for memory T cells, since their expression does not predict long time survival and rapid effector function upon secondary exposure *per se* (54). In addition, it has been shown that CD45RO^+^ T cells can be reprogrammed and go back to a CD45RO^−^ naive phenotype (55, 56). So far there are no other reliable markers of phenotype memory T cells in clinical experiments, therefore, phenotypic characterization of the memory cell population by CD45RO expression is widely used. Memory CD4^+^ and CD8^+^ cells can be divided into subsets based on their migration pattern, cytokine secretion abilities, and protein expression profile. The main memory cell subsets are the central memory (CM) cells and the effector memory (EM) cells, although the number of subsets is expanding rapidly (Tables 1, 2). The CM cell subset differentiates into effector cells upon secondary antigen exposure and is characterized by CCR7 expression which makes them home to secondary lymphoid organs (53, 57). The EM cell subset is characterized by their presence in peripheral tissue and direct pro-inflammatory effector function upon secondary antigen encounter with the cognate antigen (53). Below, an overview of the current knowledge of the various memory T cell subsets in pregnancy is reviewed (Supplementary Material).

**Table 1:** CD4^+^ memory T cells in pregnancy.

**Memory T cell**		**Markers (human)**	**Cytokines**	**Findings in pregnancy**	**Findings in complications of pregnancy**
**CD4**^**+**^	EM	CD45RO^+^, CD45RA^−^, CD44^+^, CCR7^−^, CD62L^−^, CD28^+^	IFN-gamma^+^, TNF^+^, IL4^+^, IL5^+^	- Higher proportion in peripheral blood in pregnancy (23, 30) - Higher proportions in decidua compared to peripheral blood (19, 31) - Increased IFN-gamma, IL4, PD-1, Tim-3, CTLA-4, and LAG-3 expression in decidua compared to peripheral blood (19) - Lower PD-1 expression in peripheral blood in pregnancy (23) - Higher proportion and higher activated proportion in peripheral blood postpartum compared to nulliparous women (30)	- Comparable proportions in peripheral blood in preeclampsia and healthy controls (32) - Higher proportions in peripheral blood in women with recurrent miscarriages compared to healthy controls (not specified CD4/CD8) (33)
	TEMRA	CD45RO^−^, CD45RA^+^, CCR7^−^, CD62L^−^, CD28^−^	Perforin^+^, granzyme B^+^	Not studied in pregnancy	Not studied in complications of pregnancy
	CM	CD45RO^+^, CD45RA^−^, CD44^+^, CCR7^+^, CD62L^+^, CD28^+^	IL2^+^, IFN-gamma^−^, and TNF^−^	- Higher proportion in peripheral blood compared to menstrual blood (31) - Comparable proportions and HLA-DR and CD38 expression in peripheral blood in non-pregnant and pregnant women (23, 30, 34) - Higher proportions in decidua compared to peripheral blood (31) - Higher proportion and higher activated proportion in peripheral blood postpartum compared to nulliparous women (30)	- Higher proportion in peripheral blood in preeclampsia compared to healthy controls (32) - Comparable CD27, CD28, and CD127 expression in peripheral blood in preeclampsia and healthy controls (32) - Higher proportions in peripheral blood in women with recurrent miscarriages compared to healthy controls (not specified CD4/CD8) (33, 35)
	TRM	CD45RO^+^, CD45RA^−^, CCR7^−^, CD62L^−^, CD69^+/−^, CD103^+/−^	IFN-gamma^+^, IL17^+^	Not studied in pregnancy	Not studied in complications of pregnancy
	Treg memory	CD45RO^+^, CD45RA^−^, CD44^+^, CD25^+^, CD127^−^, Foxp3^+^, CTLA4^+^	IL10^+^, TGFB^+^	- Higher proportions in the decidua compared to peripheral blood (36) - Strong increase in peripheral blood in the first trimester (37) - Comparable proportions in peripheral blood in 3rd trimester and non-pregnant women (34) - Higher immune regulating capabilities upon stimulation with umbilical cord blood (36) - HLA-DR^+^ proportion in pregnancy showed decreased suppressive activity compared to HLA-DR^+^ proportion in non-pregnant women (37) - Differentiated from RTE Treg cells in first trimester peripheral blood (38) - Remain high during pregnancy, decreased with onset of labor, and at preconception levels postpartum (38) - In mice, paternal-specific Treg memory is generated in gestation, remaining at lower levels postpartum, and lowering resorption rates in a subsequent pregnancy (12, 39, 40)	- Higher proportion in peripheral blood in preeclampsia compared to healthy controls (32) - Higher proportion of RTE Treg cells in peripheral blood in preeclampsia differentiate into CD31^+^ Treg memory cells (38). - CD31^+^ Treg memory cells in peripheral blood in preeclampsia have decreased immune suppressive capacity compared to CD31^+^ memory Treg cells in healthy women (38). - HLA-DR^−^ memory Treg cells were increased in gestational diabetes with dietary adjustment (41) - HLA-DR^−^ memory Treg cells were strongly increased in gestational diabetes with insulin therapy (41)
	FHM	CXCR5^+^, CD45RA^−^, CD45RO^+^, CD62L^+^, CCR7^+^, FR4^+^	IL21^+^, IL10^+^	- Increased in the uterus and placenta toward late gestation in mice (42)	- Higher PD-1^+^CCR7^+^ and PD-1^+^ICOS^+^ proportions in decidual tissue but not in peripheral blood from spontaneous miscarriages compared to elective terminations in healthy controls (43)
	Memory stem cell	CD45RO^−^, CD45RA^+^, CCR7^+^, CD62L^+^, CD28^+^, CD27^+^, CD95^+^, IL2RB^+^	IFN-gamma^−^, IL2^+/−^	Not studied in pregnancy	Not studied in complications of pregnancy

*EM, effector memory; TEMRA, effector memory CD45RA revertant; CM, central memory; TRM, tissue resident memory; Treg, T regulatory; FHM, follicular helper memory; CCR7, CC-chemokine receptor 7; CD62-L, L-selectin; Foxp3, forkhead box P3; CXCR5, chemokine receptor type 5; FR4, folate receptor-4; IFN-gamma, interferon-gamma; TNF, tumor necrosis factor; IL, interleukin; TGFB, transforming growth factor B; PD-1, programmed death-1; Tim-3, T cell immunoglobulin and mucin domain 3; CTLA-4, cytotoxic T lymphocyte antigen; LAG-3, lymphocyte activation gene 3; HLA-DR, Human Leukocyte Antigen-DR; ICOS, inducible T cell co-stimulator; RTE, recent thymic emigrant*.

**Table 2:** CD8^+^ memory T cells in pregnancy.

**Memory T cell**			**Markers (human)**	**Cytokines**	**Findings in pregnancy**	**Findings in complications of pregnancy**
**CD8**^**+**^	EM	General	CD45RO^+^, CD45RA^−^, CD44^+^, CCR7-, CD62L-	Combination of cytokines produced by EM1, EM2, EM3, and EM4	- Higher proportions in peripheral blood postpartum compared to nulliparous women (30) - Higher proportion in decidua compared to peripheral blood (19, 22, 31) - Comparable proportions and PD-1^+^ and PDL-1^+^ proportions in peripheral blood in pregnant and non-pregnant women (30, 34) - Higher CD38 and HLA-DR expression in peripheral blood in 3rd trimester compared to non-pregnant women (34, 44) - Higher proportions of IFN-gamma^+^ and IL4^+^ in the decidua compared to peripheral blood (19, 22) - Higher expression of PD-1, Tim-3, CTLA-4, and LAG-3 in the decidua compared to peripheral blood (19, 45, 46) - Elevated gene expression in genes involved in chemotaxis, co-inhibitory receptors, T cell activation, galectin 1, and the IFN-gamma pathway, in decidua compared to peripheral blood (19, 46) - Comparable gene expression in decidua in 1st and 3rd trimester (46)	- Comparable proportions in peripheral blood in preeclampsia and healthy controls (32) - Higher proportions in peripheral blood in women with recurrent miscarriages compared to healthy controls (not specified CD4/CD8) (33)
		EM1	CD45RO^+^, CD45RA^−^, CD44^+^, CCR7^−^, CD62L^−^, CD27^+^, CD28^+^, CD127^+^	Granzyme K^+^, Granzyme B^−^, Perforin ^+^/^−^, IFN-gamma^+^, IL4^+^, IL5^+^	- Higher proportions in the decidua compared to peripheral blood (19, 22)	- Comparable proportions in peripheral blood in preeclampsia and healthy controls (32) - Lower proportions in pregnant CMV+ women compared to pregnant CMV- women (44)
		EM2	CD45RO^+^, CD45RA^−^, CD44^+^, CCR7^−^, CD62L-, CD27^+^, CD28^−^, CD94^+^	Perforin^+/−^,Granzyme B^+/−^, IFN-gamma^+^	^−^ Higher proportions in the decidua compared to peripheral blood (19, 22) Lower proportion of perforin^+^ and granzyme B^+^ in the decidua compared to peripheral blood (22, 47)	- Comparable proportions in peripheral blood in preeclampsia and healthy controls (32)
		EM3	CD45RO^+^, CD45RA^−^, CD44^+^, CCR7^−^, CD62L^−^, CD27^−^, CD28^−^, CD94^+^	Perforin^+^, Granzyme B^+^, IFN-gamma^+^	- Higher proportions in the decidua compared to peripheral blood (19, 22) Lower proportion perforin^+^ and granzyme B^+^ in the decidua compared to peripheral blood (22, 47)	- Comparable proportions in peripheral blood in preeclampsia and healthy controls (32) - Higher proportions in pregnant CMV+ women compared to pregnant CMV- women (44)
		EM4	CD45RO^+^, CD45RA^−^,CD44^+^, CCR7^−^, CD62L^−^, CD27^−^, CD28^+^, CD127^+^	Granzyme K^+^, Granzyme B^−^, • Perforin ^+/−^, •IFN-gamma^+^	- Higher proportions in the decidua compared to peripheral blood (19, 22)	- Comparable proportions in peripheral blood in preeclampsia and healthy controls (32) - Higher proportions in pregnant CMV+ women compared to pregnant CMV- women (44)
		TEMRA	CD45RO^−^, CD45RA^+^, CCR7^−^, CD62L^−^, CD28^−^		- Higher proportions in the decidua compared to peripheral blood (22) Higher CD38 expression in peripheral blood in pregnancy compared to non-pregnant women (44)	- Higher proportions in pregnant CMV+ women compared to pregnant CMV- women (44)
	CM		CD45RO^+^, CD45RA^−^, CD44^+^, CCR7^+^, CD62L^+^, CD28^+^, CD27^+^, CD127^+^	Perforin^−^, Granzyme B^−^, IFN-gamma^−^, IL2^+^	- Low proportions in decidua, peripheral blood and menstrual blood (22, 31) - Higher proportions in peripheral blood compared to menstrual blood (31) - Comparable proportions and CD38^+^, CD28^+^, and CD27^+^ proportions in peripheral blood in pregnant and non-pregnant women (23, 30, 34) - Higher HLA-DR^+^ expression in peripheral blood in 3^rd^ trimester compared to non-pregnant women (34)	- Comparable CD28^+^ proportions in peripheral blood in preeclampsia and healthy controls (32) - Higher proportions in peripheral blood in women with recurrent miscarriages compared to healthy controls (not specified CD4/CD8) (33)
	TRM		CD45RO^+^, CD45RA^−^, CD103^+^/^−^, CD69^+^, CCR7^−^, CD62L^−^, CD49A^+^	Granzyme B^+^, IFN-gamma^+^, perforins^+^	- Present in the reproductive tract (48, 49) - In the reproductive tract do not require IL15 for maintenance and work independently from CD4^+^ cells (48–50)	- Comparable proportions in endometrial tissue from women with recurrent miscarriages compared to healthy controls (51)
	Treg memory		Unknown	Unknown	- Not studied in pregnancy	- Not studied in pregnancy
	FHM		CD45RO^+^, CXCR5^+^	IL21^+^, IL4^+^, IFN-gamma^+^	- Not studied in pregnancy	- Not studied in pregnancy
	Memory stem cell		CD45RO^−^, CD45RA^+^, CCR7^+^, CD62L^+^, CD28^+^, CD27^+^, CD95^+^,	IFN-gamma^+^, IL2^+^	- Not studied in pregnancy	- Not studied in pregnancy

*EM, effector memory; TEMRA, effector memory CD45RA revertant; CM, central memory; TRM, tissue resident memory; Treg, T regulatory; FHM, Follicular Helper Memory; CCR7, CC-chemokine receptor 7; CD62-L, L-selectin; CXCR5, chemokine receptor type 5; IFN-gamma, interferon-gamma; IL, interleukin; PD-1, programmed death-1; PDL-1, programmed death ligand-1; Tim-3, T cell immunoglobulin and mucin domain 3; CTLA-4, cytotoxic T lymphocyte antigen; LAG-3, lymphocyte activation gene 3; CMV, cytomegalovirus; HLA-DR, human leukocyte antigen-DR*.

## CD4^+^ Memory Cells in Pregnancy

Within the CD4^+^ memory cell population, a subdivision has been made based on migration pattern and effector function; i.e., CD4^+^ effector memory (CD4^+^ EM) cells, CD4^+^ central memory (CD4^+^ CM) cells, CD4^+^ tissue resident memory (CD4^+^ TRM) cells, CD4^+^ T follicular helper memory (CD4^+^ FHM) cells, CD4^+^ regulatory memory cells, and CD4^+^ memory stem cells (58–65).

It has been known for many years that pregnancy and some pregnancy complications affect the general CD4^+^ memory T cell population. In 1996, it was shown that general CD4^+^ memory cell (CD4^+^CD45RO^+^) proportions in peripheral blood were lower from the second trimester onwards until 2–7 days postpartum compared to proportions in non-pregnant controls (66). These findings have been followed up by studies in preeclampsia (67–69), gestational diabetes (70), and preterm labor (71) in which higher proportions of total memory T cells in peripheral blood have been found compared to healthy pregnant controls. Early studies also showed CD4^+^CD45RO^+^ memory cells in the decidua and showed that CD45RO expression on CD4^+^ cells is upregulated in the decidua compared to CD4^+^ cells in peripheral blood (72, 73). Later, Gomez-Lopez et al. suggested a role for CD4^+^ memory cells in human term parturition by showing an increase of CD4^+^ memory cells (CD4^+^CD45RO^+^) using immunohistochemistry on choriodecidual tissue from women in spontaneous labor at term compared to women with term scheduled cesarean sections (74). The early data already indicated that memory T cells are affected by pregnancy and its complications. In more recent years, studies have focused on specific subsets of CD4^+^ memory cells. These data are reviewed per memory T cell subset below.

### CD4^+^ Effector Memory Cells in Pregnancy

Th1, Th2, and possibly Th17 effector cells can differentiate into CD4^+^ EM cells (75–77). CD4^+^ EM cell characterization is based on the lack of expression of lymph node homing receptors CC-chemokine receptor-7 (CCR7) and CD62L (L-selectin), which enables them to migrate to peripheral tissue (59). EM cells are the memory cells with the fastest immune response on a secondary encounter. Within several hours after re-stimulation with a memorized antigen, CD4^+^ EM cells produce a variety of cytokines as interferon-gamma (IFN-gamma), tumor necrosis factor (TNF), interleukin-4 (IL4), and IL5 (53, 77, 78). A specific subtype of CD4^+^ EM cell can re-express CD45RA after antigen stimulation (TEMRA) (79). These cells are poorly studied and there are no published investigations on CD4^+^ TEMRA cells in reproduction to our knowledge.

In the second and third trimesters of pregnancy, two studies showed higher CD4^+^ EM cell (CD45RA^−^CCR7^−^ and CD45RO^+^CCR7^−^) proportions in peripheral blood, compared to proportions of these cells in non-pregnant women (23, 30), while another study found decreased numbers of CD4^+^ EM cells in peripheral blood during pregnancy (34). Differences between the studies could be due to the fact that that hormonal fluctuations during the menstrual cycle were not taken into account in the latter study. Not only is the proportion of CD4^+^ EM cells increased during pregnancy, these cells also showed increased expression of CD69 (30), as well as decreased expression of programmed death-1 (PD-1) (23). This suggests that there is increased activation of CD4^+^ EM cells, and that these cells are less susceptible to apoptosis. The increase of CD4^+^ EM cells is not only seen during pregnancy, but also years after when CD4^+^ EM cell proportions remained increased, i.e., at gestation levels as compared with women that have never been pregnant (30). These cells also showed increased CD69 expression after pregnancy, which could suggest persistent activation through exposure to antigen. This could be related to microchimerism, although it remains to be investigated whether the increased EM cell proportion is due to an increase in cells specific for paternal-fetal antigens.

Whereas, in blood the proportion of CD4^+^ EM cells of the total CD4^+^ cell population was about 20–30% (19, 30, 31), locally, in the decidua, the proportion of CD4^+^ EM cells (CD45RA^−^CCR7^−^) was higher with 50–60% of the total CD4^+^ cell population being EM cells (19, 31). This may indicate accumulation of CD4^+^ EM cells in the decidua, although it can also be simply due to the fact that naive T cells do not accumulate in peripheral tissue (80). Important for the function of memory T cells is the expression of co-stimulatory molecules like CD28 (81). Such molecules are important for the recall response of memory T cells (82). Interestingly, within the CD4^+^ EM cell population in the decidua, the proportion of the EM subset not expressing co-stimulatory molecules is highly increased compared to peripheral blood (19), suggesting that the CD4^+^ EM cells in the decidua may not be able to mount a secondary response comparable to CD4^+^ EM cells in peripheral blood. Despite this, increased IFN-gamma and IL4 expressions were found in decidual CD4^+^ EM cells compared to CD4^+^ EM cells in peripheral blood *in vitro* following mitogen stimulation (19). This may be related to the high local progesterone concentrations at the fetal maternal interface (19). The decidual EM cells were not only able to respond to mitogen stimulation, they were also able to respond to fetal antigens (19). The fact that the decidual EM cells are able to respond to fetal antigens and other stimuli suggests that there are extrinsic or intrinsic mechanisms at the fetal-maternal interface to suppress these cells. One of these mechanisms could be the presence of Treg cells (83, 84). Another mechanism may be the expression of immune inhibitory checkpoint receptors on decidual CD4^+^ EM cells (19). Activation of these receptors inhibit immune responses to avoid autoimmunity and chronic inflammation (85). Increased expression of the immune inhibitory checkpoint receptors PD-1, T cell immunoglobulin and mucin domain 3 (Tim-3), cytotoxic T lymphocyte antigen 4 (CTLA-4), and lymphocyte activation gene 3 (LAG-3), on CD4^+^ EM cells in the decidua was found as compared to peripheral blood (19). These findings are in line with Wang et al. who showed that the majority of CD4^+^ EM cells (CD44^+^CD62L^−^) in first trimester decidual tissue from healthy terminated human pregnancies, expressed Tim-3 and PD-1 (86). A role for such immune inhibitory check point receptors in pregnancy has been shown in mouse studies (86). Blocking the Tim-3 and PD-1 pathway (not on CD4^+^ EM cells specifically) in healthy pregnant mice showed that lower expression of Tim-3 and PD-1 increased fetal resorption rates (86). These studies propose a regulatory function for CD4^+^ EM cells locally that could be favorable for fetal-maternal immune tolerance and prevent pregnancy loss.

The current data on CD4^+^ EM cells in women with uncomplicated pregnancy outcomes show that during pregnancy CD4^+^ EM cells may accumulate in the decidua and remain present at higher levels and higher activated proportions in peripheral blood postpartum (30). In addition, the CD4^+^ EM cell population in the decidua has a different phenotype with increased IFN-gamma expression, however the CD4^+^ EM cell population also has increased expression of immune inhibitory proteins compared to peripheral blood (19). To understand the relevance and function of CD4^+^ EM cells in fetal-maternal tolerance and their role in the postpartum period, further research should focus on their general and more specifically on their antigen specific function, since none of the studies has shown antigen specific tolerance induction by CD4^+^ EM cells yet.

Unfortunately, until now, CD4^+^ EM cells have been hardly studied in complications of pregnancy. CD4^+^ EM cells were studied in preeclampsia by Loewendorf et al. who performed flow cytometric analyses on peripheral blood and a swab from the intrauterine cavity during cesarean sections (32). They did not find differences in levels of CD4^+^ EM cells between healthy and preeclamptic women in peripheral blood or in lymphocytes isolated from the intra uterine swab (32). However, since the specific tissue of origin of the cells from the swab cannot be defined, caution should be taken when interpreting these results. In non-pregnant women suffering recurrent spontaneous miscarriages, higher proportions of EM cells were observed in peripheral blood compared to non-pregnant fertile controls (33). This study did not further specify the CD4^+^ or CD8^+^ status or phenotype. With the proposed relevance of CD4^+^ EM cells in fetal-maternal tolerance it would be of great value to gain knowledge on CD4^+^ EM cells in complications of pregnancy.

### CD4^+^ Central Memory Cells in Pregnancy

CD4^+^ CM cells circulate in the blood and are home to lymph nodes through expression of lymph node homing receptors CCR7 and CD62L (57–59). CD4^+^ CM cells secrete IL2 and only very low levels of effector cell cytokines (28, 53). Upon secondary antigen exposure, or spontaneously, in the presence or absence of polarizing cytokines, CD4^+^ CM cells differentiate into Th1, Th2, and CD4^+^ EM cells, and produce effector cytokines as IFN-gamma and IL4 (53, 87–89). Furthermore, CM cells can quickly cause expansion of the antigen specific T cell population (89).

During pregnancy, as for CD4^+^ EM cells, CD4^+^ CM cells are studied mainly in the circulating blood and less at the fetal-maternal interface. One study looked at CD4^+^ CM cells (CD45RA^−^CCR7^+^) in decidual tissue at the end of pregnancy and showed that proportions of CD4^+^ CM cells were higher compared to peripheral blood from non-pregnant women (31). Another study evaluated first trimester decidual tissue from terminated healthy pregnancies, and showed that about 40% of CD4^+^ CM cells (CD44^+^CD62L^+^) were Tim-3^+^ and PD-1^+^ (86). This appears to be a subset of CD4^+^ EM cells that have a strong suppressive capacity on proliferation and preferentially produce Th2 type cytokines (86). Since blocking of PD-1 and Tim-3 in pregnancies in mice induced fetal loss (86), the Tim-3^+^PD-1^+^ CD4^+^ EM cells may be important for maintaining normal pregnancy.

A number of studies in pregnancy observed that the proportions of CD4^+^ CM cells (CD45RA^−^CCR7^+^) in peripheral blood are comparable between women in the second or third trimester of pregnancy and in healthy non-pregnant women (23, 30, 34). However, it seems that after pregnancy the CD4^+^ CM cell proportions in peripheral blood are increased, since CD4^+^ CM cells were higher in women after pregnancy compared to pregnant women and compared to women that have never been pregnant (30). Whether the CD4^+^ CM cells are activated in the circulation of pregnant women remains to be established, since expression of the activation marker CD69 was higher during pregnancy as compared with non-pregnant women (30), whereas expression of the activation markers HLA-DR and CD38 was not affected in the CD4^+^ CM cell population (CCR7^+^CD45RO^+^) in peripheral blood from 3rd trimester pregnant women compared to non-pregnant women (34). This higher CD69^+^ proportion of CD4^+^ CM cells in pregnancy remained high in women after pregnancy compared to women who have never been pregnant (30).

To date, CD4^+^ CM cells are investigated in two complications of pregnancy, i.e., preeclampsia and miscarriages. In preeclampsia, slightly, but significantly higher proportions of CD4^+^ CM cells (CD45RO^+^CCR7^+^) were found in peripheral blood from preeclamptic women compared to healthy pregnant women (32). Proportions of CD4^+^ CM cells isolated from a swab from the intrauterine cavity during a cesarean section did not show differences between preeclamptic and healthy pregnant women (32). This study also analyzed expression of co-stimulatory molecules, CD28, CD27, and the survival receptor CD127 (IL7 receptor alpha chain), on CD4^+^ CM cells (32). Only a difference in CD28 expression was found: in an intrauterine swab from preeclamptic women, CD4^+^ CM cells expressed lower levels of CD28 compared to healthy pregnant women (32). In peripheral blood this difference was not observed (32). In women suffering from recurrent spontaneous miscarriages, higher levels of CM cells (CD45RO^+^CD62L^+^) have been found in peripheral blood compared to fertile women (33). It was not specified whether these CM cells were from the CD4^+^ or the CD8^+^ lineage. Part of this increase is likely due to CD4^+^ CM cells, since another study reported higher levels of CD4^+^ CM cells (CD4^+^CD45RA^−^CCR7^+^) in peripheral blood from women suffering from recurrent miscarriages compared to women with proven fertility and women with no previous pregnancies (35).

As indicated above, Tim-3 and PD-1 expression on memory cells may be important for a healthy pregnancy. This suggestion is in line with the finding of decreased proportions of Tim-3^+^PD-1^+^ CD4^+^ cells in decidua from patients who had undergone miscarriage (86). Unfortunately, these CD4^+^ cells were not stained for memory cell markers. Further studies on CD4^+^ CM cells in pregnancy complications in blood and at the fetal-maternal interface are needed in order to be able to show that these cells may play a role in the physiology of pregnancy and the pathophysiology of complications.

### CD4^+^ Regulatory Memory Cells in Pregnancy

Treg cells have potent immunosuppressive properties. They produce IL10 and transforming growth factor beta (TGFB), and have the capability of suppressing CD4^+^, CD8^+^, and B cell proliferation and cytokine secretion as well as inhibiting effects on dendritic cells and macrophages (90–93). It was long assumed that Treg cells did not survive the contraction phase of the immune response and undergo apoptotic cell death (64). Nevertheless, a long time surviving memory Treg cell subset has now been shown to persist after antigen exposure (12, 64, 94, 95). There is increasing evidence that memory Treg cells regulate the EM immune response on a secondary encounter with a memorized antigen (64). Treg memory function is implicated in many different pathological and physiological contexts such as auto-immune diseases (96), respiratory disorders (97), hepatitis (98), and pregnancy (12). Treg memory cells are complex to study, since no conclusive markers for a long-living Treg cell population are known (64). Identification of the Treg memory cell pool is therefore performed by combining Treg cell markers as [forkhead box p3 (Foxp3^+^), CD25^+^, and CD127^−^ (99)] with memory cell markers [as CD45RO^+^ and CD45RA^−^ (52, 53)] (64).

In rodent models, Treg cells with fetal antigen specificity and a memory phenotype have been shown to accumulate in gestation and impact reproductive success in subsequent pregnancies (12, 39, 40). Rowe et al. developed a mouse model that demonstrated an increase of fetal antigen specific Treg memory cells at mid-gestation in first pregnancies that remained present at lower levels postpartum (12). The Treg memory cell population expanded substantially with accelerated kinetics in a following pregnancy as compared with the first pregnancy (12). This expansion resulted in decreased resorption rates compared to Treg memory cell ablated mice (12). The fetal antigen specific memory Treg cells, as shown by Rowe et al. seem important at mid gestation and it is hypothesized that they might be especially valuable in subsequent pregnancies to set boundaries for a secondary EM cell response toward paternal-fetal antigens (12). Chen et al. showed that in early gestation in mice (during implantation) self-antigen specific memory Treg cells and not fetal antigen specific memory Treg cells are recruited to the reproductive tract and create the tolerant environment for the implantation of the blastocyst (39).

Similar to mouse studies, in early pregnancy fetal maternal immune tolerance is probably not exclusively managed by memory Treg cells, since higher naive Treg cell subsets were associated with successful *in vitro* fertilization (IVF)/intracytoplasmic sperm injection (ICSI) treatment (37). Schlossberger et al. distinguished naive Treg (CD45RA^+^CD25^+^Foxp3^+^) and memory Treg (CD45RA^−^CD25^+^Foxp3^+^) subsets in blood samples from women undergoing IVF/ICSI treatment and observed higher proportions of naive Treg cells in women who became pregnant compared to the women who did not (37). This finding could indicate that in (preparation for) early pregnancy, higher levels of naive Treg cells are important for successful pregnancy. It could be speculated that these higher levels of naive Treg cells might be able to proliferate into antigen experienced memory Treg cells which are possibly beneficial in late pregnancy. This hypothesis needs to be tested in further studies, but would be in line with findings in mouse studies, in which the paternal antigen specific Treg memory cells were important at mid gestation (12).

The same group followed up on this study and showed that in healthy pregnant women, in early pregnancy (1st trimester) the decrease in naive Treg cells is most likely due to a decreased output of thymic Treg cells, since a decrease in recent thymic emigrant Treg cells was found in early pregnancy (38). They also showed that the increase in memory Treg cells in early pregnancy seems to be due to a differentiation of the recent thymic emigrant Treg cells, since an increased proportion of CD45RA^−^CD31^−^ memory Treg cells was found (38), which returned to normal non-pregnancy levels over the course of pregnancy (38). In line with their previous publication (37), this group showed again that the suppressive capacity of the naive Treg cells is increased during pregnancy and the suppressive capacity of the memory Treg cell population is decreased during pregnancy (38). At the end of pregnancy, the proportion of CD4^+^ Treg memory cells (CD45RA^−^Foxp3^+^) in peripheral blood were present at comparable levels as in non-pregnant women (34, 38), which may suggest that memory Treg cells either undergo apoptotic cell death or reside in tissues toward the end of pregnancy. Thus, CD4^+^ memory Treg cells are found to be favorable for pregnancy in mice, differentiate from recent thymic emigrant Treg cells in early human pregnancy, and circulate in peripheral blood. Studies on their presence and function at the fetal-maternal interface during pregnancy and studies postpartum and during a second pregnancy are lacking.

Foxp3^+^ Treg cells are implicated in the pathophysiology of many complications of pregnancy as, preeclampsia (4, 100), recurrent miscarriage (101), and infertility (4, 102), however the potential role of the memory cell subset of the Treg cell population in different complications is not well studied. In preeclampsia, there was a decrease of naive Treg cells and an increase in memory Treg cells as compared with healthy pregnancy (32, 103). Although the naive Treg population in preeclampsia showed decreased suppressive activity compared with healthy pregnancy, this was not the case for the memory cell population (103). Further studies are needed to evaluate the role of the memory Treg population in preeclampsia.

In women with gestational diabetes, phenotypic characterization of memory Treg cell subsets showed that the proportion of naive Treg cells (CD45RA^+^HLA-DR^−^CD127^+^Foxp3^+^) was lower in women with gestational diabetes compared to healthy pregnant women, independently of whether diabetes was treated with a diet or insulin (41). The proportion of memory Treg cells, on the other hand, increased in gestational diabetes (41). Within the memory Treg cell population HLA-DR^+^ and HLA-DR^−^ memory Treg cells are distinguished (104), in which HLA-DR^+^ memory Treg cells have a more differentiated phenotype, are more suppressive and secrete lower amounts of pro-inflammatory cytokines as compared with HLA-DR^−^ memory Treg cells (104). Whereas, HLA-DR^−^ memory Treg cells were increased in gestational diabetes with dietary adjustment, HLA-DR^+^ memory Treg cells were strongly increased in gestational diabetes treated with insulin therapy compared to healthy pregnant women (41). Whether this is an effect of the insulin treatment or a reflection of the pathophysiology of the disease is not known.

In summary, studies on memory Treg cells in complications of pregnancy show that memory Treg cells might be beneficial for reproductive success in subsequent pregnancies in mice (12), however human studies are inconclusive so far. The fact that some studies find higher memory Treg cell levels in pregnancy complications such as preeclampsia and gestational diabetes (32, 41, 103), whereas others find that lower levels prior to embryo transfer in IVF/ICSI treatment are associated with pregnancy success (37), could indicate specific roles depending on the phase of pregnancy. Identification of more conclusive markers for memory Treg cell function and longevity is a priority to fully elucidate the role of memory Treg cells in reproduction. In addition, since previous studies were mostly performed in peripheral blood, studies on memory Treg cells should also focus on the fetal-maternal interface, as it is known that memory T cells not only reside in peripheral tissues but also in the decidua (48, 74).

### CD4^+^ Follicular Helper Memory Cells in Pregnancy

CD4^+^ follicular helper cells, which are located mainly in lymphoid organs and in particular in the germinal centers of lymphoid organs, also have a memory cell subset, called CD4^+^ FHM cells (63, 105). They are known to assist B cells in their differentiation process and produce IL10 and IL21 (106). CD4^+^ FHM cells are recognized by CXCR5, CD62L, CCR7, and Folate receptor 4 (FR4) (106). Contrary to the effector T follicular helper subset, CD4^+^ FHM cells exhibit low B-cell lymphoma 6 (Bcl-6) expression (63, 106, 107). Bcl-6 is a transcriptional suppressor of GATA3, TBET, and RORGT, and is of major importance for T follicular helper functioning and maintenance (63). Within the CD4^+^ FHM cell population, different CD4^+^ FHM cell subsets can be distinguished based on PD-1, CCR7, and inducible T cell co-stimulator (ICOS) expression (63, 106, 107).

One mouse and one human study reported on CD4^+^ FHM cells in pregnancy (42, 43). In mid gestation, in mice after allogeneic mating, T follicular helper cells (CD4^+^CXCR5^+^PD-1^+^/ICOS^+^) were shown to accumulate in the uterus and placenta (42). These CD4^+^ T follicular helper cells could be CD4^+^ FHM cells, since they showed an activated memory (CD44^+^) phenotype. This putative CD4^+^ FHM population increased abundantly toward late gestation, but this study also showed that programmed death ligand-1 (PDL-1) blockage induced abortion and increased the putative CD4^+^ FHM cell accumulation even further (42). The study does suggest that CD4^+^ FHM cells may be implicated in fetal-maternal tolerance and that excessive abundance might be associated with pregnancy loss (42).

In accordance with the suggestion that increased numbers of CD4^+^ FHM cells may be implicated in pregnancy loss, a human study in recurrent miscarriage found higher decidual CD4^+^ FHM cells (CXCR5^+^PD-1^+^CCR7^−^ and CXCR5^+^PD-1^+^ICOS^+^) in spontaneous miscarriage decidual tissue compared to tissue from elective terminations in healthy women (43). In peripheral blood, the proportions of CD4^+^ FHM cells (CXCR5^+^PD-1^+^CCR7^−^ and CXCR5^+^PD-1^+^ICOS^+^) did not differ between the groups, implying a local response (43). In summary, the current data that exist on CD4^+^ FHM cells in complications of pregnancy may suggest that pregnancy loss is associated with abundance of CD4^+^ FHM cells. Thorough research is necessary to increase fundamental knowledge on the function of TFH memory cells in normal and complicated pregnancies.

### CD4^+^ Tissue Resident Memory Cells in Pregnancy

In the classification of memory T cells, CD4^+^ TRM cells are distinguished from circulating cells (108, 109). Since no conclusive defining markers for the TRM cells from the CD4^+^ compartment are known, they are difficult to investigate (62, 108, 109). Occasionally, the markers for CD8^+^ TRM cells are used to study TRM cells in the CD4^+^ compartment, although it is unclear whether this is correct (Table 2) (108, 109). To the best of our knowledge, no literature on CD4^+^ TRM cells in reproduction is published yet.

### CD4^+^ Memory Stem Cells in Pregnancy

CD4^+^ memory stem cells are a rare kind of memory T cell that cannot be classified according to the general differentiation of naive and memory cells using the CD45 isoforms (65). Long living cells with a naive phenotype (CD45RA^+^CCR7^+^CD27^+^), but with antigen specificity and effector function, were shown in human blood years after Epstein Barr Virus infection (110). The so-called T memory stem cells exhibit almost all conventional memory cell like properties as high CXCR3, CD95, and IL2 receptor beta expression (65, 111), however they lack CD45RO expression and show similar recirculation patterns as naive T cells (65). Studies have shown that CD4^+^ memory stem cells play a role in auto-immune diseases, Human Immunodeficiency Virus (HIV) and immune protection from a range of infections (65). To our knowledge, CD4^+^ memory stem cells have not been studied in reproduction yet.

## CD8^+^ Memory Cells in Pregnancy

Similar to CD4^+^ memory cells, CD8^+^ memory cell subsets are distinguished according to their migration pattern, cytokine secretion abilities, and protein expression (Table 2) (112–114). CD8^+^ memory cells are divided in several subpopulations: CD8^+^ effector memory cells (CD8^+^ EM), CD8^+^ central memory cells (CD8^+^ CM), CD8^+^ tissue resident memory cells (CD8^+^ TRM), CD8^+^ follicular helper memory cells (CD8^+^ FHM), and CD8^+^ memory stem cells (58–65, 115, 116). CD8^+^ memory cells with regulatory properties are described, however there is no consensus on existence of a CD8^+^ Treg memory subset (45). Most CD8^+^ memory cells are generated from antigen experienced effector cells over the course of an immune response (113, 117, 118), however some CD8^+^ memory cells may arise directly from naive T cells (119, 120). CD8^+^ memory cells form the first line of defense in mucosal tissues and are able to produce effector cytokines and granzymes, IFN-gamma and perforin upon stimulation without the need for co-stimulatory signals (53, 121).

It has been known for many years that CD8^+^ memory cells are present in the decidua during pregnancy (73). Higher CD45RO^+^ proportions of CD8^+^ cells were found in first trimester decidua compared to peripheral blood at the same time of pregnancy (72, 73). Furthermore, the proportion of CD8^+^ memory (CD8^+^CD45RO^+^) cells in peripheral blood did not differ between pregnant and non-pregnant women (30, 73). In a further study, the CD8^+^ memory T cell population was found to be influenced by seminal fluid (122). Using immunohistochemistry, CD8^+^ memory cells (CD3^+^CD8^+^CD45RO^+^) were shown to be increased in the stroma and epithelium of human cervix biopsies taken 12 h after unprotected coitus compared to biopsies after a period of abstinence and biopsies after coitus with condom use (122). Although this shows that memory CD8^+^ cells are generated as a response toward seminal fluid, it is unknown whether these cells are specific to the paternal antigen. Furthermore, their role in preparation for pregnancy and fetal-maternal tolerance is not known. More recent studies have focused on evaluating the different CD8^+^ memory cell subsets in reproduction. This is reviewed per subset below.

### CD8^+^ Effector Memory Cells in Pregnancy

CD8^+^ EM cells express CD45RO, but lack CCR7 expression and are therefore bound to circulate in peripheral blood and non-lymphoid tissue (28, 112). CD8^+^ EM cells rapidly produce effector cytokines as IL4, IL5, and IFN-gamma upon secondary encounter with the cognate antigen and therewith generate immediate protection (24). The CD8^+^ EM cells express co-stimulatory molecules CD27 and CD28, which are gradually lost with differentiation of CD8^+^ EM cells (47). Using these molecules, the CD8^+^ EM cell population can be subdivided in 4 EM cell subtypes; i.e., EM1 (CD27^+^CD28^+^), EM2 (CD27^+^CD28^−^), EM3 (CD27^−^CD28^−^), and EM4 (CD27^−^CD28^+^) (Table 2) (47), with EM-1 being the most prominent in peripheral blood (about 70%) (47, 123). Next to a different immune phenotype, these subsets may exert different functions (47).

CD45RA expression on CD8^+^ T cells is widely known to be lost on antigen exposure, however on one highly differentiated subpopulation of CD8^+^ EM cells, CD45RA is again expressed despite previous antigen exposure (47, 121). These CD8^+^ memory cells are terminally differentiated and called CD45RA revertant effector memory cells (CD8^+^ TEMRA or sometimes abbreviated EMRA) (47, 121). CD8^+^ TEMRA cells exhibit great cytolytic activity, but lack expansion abilities and CCR7 expression, disabling them to migrate to secondary lymphoid tissue (47, 121).

Increasing evidence shows that CD8^+^ EM cells are involved in the establishment of functional immune tolerance toward the fetus (20, 46, 124). In peripheral blood, the total CD8^+^ EM cell population was similar in healthy non-pregnant women compared to healthy pregnant women in the 2nd and 3rd trimesters (30, 34). Several studies showed an altered activation marker profile on CD8^+^ EM cells in pregnancy. Higher expression of CD38^+^ on CD8^+^ EM (CD45RO^+^CCR7^−^) and CD8^+^ TEMRA (CCR7^−^CD45RA^+^) cells was found in peripheral blood from pregnant women in the 3rd trimester compared to non-pregnant women (34, 44). Moreover, higher HLA-DR expression, but comparable CD69 expression, were found on CD8^+^ EM cells (CD45RO^+^CCR7^−^) in peripheral blood in pregnant women compared to non-pregnant women (30, 34). Interestingly, although during pregnancy the proportions of CD8^+^ EM cells in blood were not different from the proportion in non-pregnant women, higher proportions of CD8^+^ EM cells (CD45RO^+^CCR7^−^) were found in peripheral blood from women postpartum compared to women who have never been pregnant (30). The higher expression of some of the activation markers on CD8^+^ EM cells in pregnancy suggest that CD8^+^ EM cells are activated in peripheral blood in pregnancy. A similar expression of inhibitory molecules PD-1 and PDL-1 on CD8^+^ EM cells was found, suggesting that their effector function remains the same (23).

Approximately half of the CD8^+^ cells in the decidua were found to be CD8^+^ EM cells (CD45RA^−^CCR7^−^), which is about two-fold higher than the proportion of these cells in peripheral blood (19, 22, 31). This may be due to preferential accumulation of these cells in the decidua, but as for naive CD4^+^ cells, it may also be due to the fact that naive CD8^+^ cells do not accumulate in peripheral tissues (80). Not only does the proportion of CD8^+^ EM cells differ between peripheral blood and the decidua, also substantial differences in phenotype, gene expression and function between these cells in peripheral blood and decidua have been observed (19, 22, 31, 46). CD8^+^ EM cells (CD45RA^−^CCR7^−^) in the decidua have shown increased IFN-gamma and IL4 secretion abilities and reduced perforin and granzyme B expression compared to CD8^+^ EM cells in peripheral blood (19, 22). Whether these specific functionalities of the decidual CD8^+^ EM cells contribute to fetal-maternal immune tolerance remains to be established.

More evidence for altered functionality of CD8^+^ EM cells in the decidua compared to peripheral blood was found by a study showing elevated expression of inhibitory check point receptors PD-1, Tim-3, CTLA-4, and LAG-3 on decidual CD8^+^ EM cells compared to CD8^+^ EM cells in peripheral blood (19, 45, 46). The higher Tim-3 and PD-1 expression on decidual CD8^+^ T cells might be the result of interaction with trophoblasts, since co-culturing CD8^+^ T cells with trophoblasts induced upregulation of Tim-3 and PD-1 (45), suggesting that trophoblasts may induce a function change, i.e., tolerance in CD8^+^ EM cells in the decidua. In accordance with the increased expression of activation markers, inhibitory check point receptors, and cytokine production in decidual CD8^+^ EM cells is the elevated gene-expression of several genes that was found in decidual CD8^+^ EM cells compared to CD8^+^ EM cells in peripheral blood (19, 46). Genes involved in chemotaxis, inhibitory receptors, T cell activation, Treg cell differentiation and genes associated with the IFN-gamma pathway were found higher in decidual CD8^+^ EM cells compared to peripheral blood CD8^+^ EM cells (19, 46). The different characteristics of decidual CD8^+^ EM cells vs. peripheral blood CD8^+^ EM cells might be beneficial for immune tolerance at the fetal maternal interface.

The question arises whether the changes in CD8^+^ EM cells are due to the appearance of fetal specific CD8^+^ EM cells. H HY tetramers are used to detect maternal T cells with specificity for Y-chromosome encoded HY-protein expressed by a male fetus (125). The proportion of HY-specific CD8^+^ cells (not further specified which memory subtype) in peripheral blood in early pregnancy was 0.035% of the CD8^+^ population, which almost tripled toward the end of pregnancy (10). The majority of the HY-specific CD8^+^ memory cell population in peripheral blood and decidua showed an effector memory phenotype, being either CD8^+^ EM (CCR7^−^CD45RA^−^) or CD8^+^ TEMRA (CD45RA^+^CCR7^−^) (10, 125). Upon stimulation with male cells, the HY-specific T cells were cytotoxic and secreted IFN-gamma (10). The HY specific CD8^+^ cells in the decidua expressed higher PD-1 and CD69 as compared with peripheral blood (19).

In preeclampsia, CD8^+^ EM cell proportions and their CD27 and CD28 expression were comparable to CD8^+^ EM cell proportions in healthy women in peripheral blood and in a swab from the intrauterine cavity (32). Contrary to preeclampsia, in non-pregnant women following recurrent spontaneous miscarriages, higher proportions of EM cells (not specified whether from the CD4^+^ or CD8^+^ cell compartment) were observed in peripheral blood compared to fertile non-pregnant controls (33). Lissauer et al. found that CD8^+^ EM cell subsets are present at different proportions in pregnancy in women with latent CMV infection (44). They found that in CMV seropositive women the proportion of CD8^+^ TEMRA cells (CD45RA^+^CCR7^−^) was higher and that the CD8^+^ EM cell population was more differentiated with higher EM3 (CD28^−^CD27^−^) and EM4 CD28^+^CD27^−^) phenotypes and lower EM1 (CD28^+^CD27^+^) compared to CMV seronegative pregnant women (44). With the proposed important role for CD8^+^ EM cells in successful pregnancies, it is worthwhile to investigate CD8^+^ EM cells and their function in peripheral blood and in the decidua in complications of pregnancy to further evaluate their role in reproduction.

### CD8^+^ Central Memory Cells in Pregnancy

CD8^+^ CM cells have little effector function and need to be converted to other cell types before effector functions can be induced (53, 112). In contrast to CD8^+^ EM cells, they are highly proliferative upon stimulation and express the lymph node homing receptor CCR7, which allows these cells to migrate to secondary lymphoid tissue (57). CD8^+^ CM cells have the ability to generate a diverse progeny, with different types of daughter cells like CD8^+^ EM cells and effector cells (126). The main cytokine produced by CD8^+^ CM cells is IL2, but they also produce low levels of IFN-gamma and TNF (112).

In reproduction, CD8^+^ CM cells are less well studied than CD8^+^ EM cells, this could be explained by their low prevalence, as the proportions of CD8^+^ cells with a CM phenotype in the decidua and peripheral blood are low (about 5% of CD8^+^ cells) (22, 30, 31). Three studies showed that CD8^+^ CM cell proportions in peripheral blood are not altered by pregnancy (23, 30, 34). CD38, CD28, and CD27 expression on the CD8^+^ CM cell population was also found to be similar in peripheral blood in pregnant and non-pregnant women (23), although HLA-DR expression on CD8^+^ CM cells was found higher in peripheral blood from women in the third trimester compared to non-pregnant women (34). Investigation of male-fetus specific CD8^+^ CM cells in peripheral blood using HY-dextramer staining, revealed that very low proportions of HY specific CD8^+^ cells have a CD8^+^ CM phenotype (10, 19). This could suggest that fetal antigens do not reach the secondary lymphoid tissue, less HY-specific CD8^+^ CM cells develop, and less HY-specific CD8^+^ CM cells recirculate into peripheral blood (10). Whether less HY-specific CD8^+^ cells develop is not known.

Whether CD8^+^ CM cells are present at different proportions in decidual tissue compared to peripheral blood remains to be established, since one study did not find differences, while another study found significantly lower CD8^+^ CM cell (CD45RA^−^CCR7^+^) proportions in decidual tissue compared to peripheral blood (22, 31). A possible explanation for the discrepancy could be methodological, as only one of the studies used a viability stain. Granzyme B and perforin are very low expressed by CD8^+^ CM cells and no differences have been found for granzyme B and perforin expression when comparing decidual and peripheral blood CD8^+^ CM cells (22).

In preeclampsia, CD8^+^ CM cell proportions and their CD28 and CD27 expression were comparable to the proportions in healthy women, both in peripheral blood and in a swab from the intrauterine cavity (32). In peripheral blood from non-pregnant women suffering from recurrent spontaneous miscarriage, higher proportions of CD8^+^ CM cells (CD45RO^+^CD62L^+^) were found compared to non-pregnant fertile women (33). However, as CD4^+^ or CD8^+^ cell phenotype was not identified, it is not sure if this finding reflects a difference in CD8^+^ CM cells.

### CD8^+^ Tissue Resident Memory T Cells in Pregnancy

Tissue resident memory (TRM) cells are a distinct subpopulation of CD8^+^ memory cells which reside in peripheral tissues, including endometrium and decidua (49, 51). After the primary immune response, CD8^+^ TRM cells reside in peripheral tissues awaiting a secondary encounter without recirculating in peripheral blood or lymph nodes (127–129). Upon reactivation, CD8^+^ TRM cells produce IFN-gamma, granzyme B, and perforins (127). TRM cells are typically identified by the expression of different surface markers as CD103, CD69, and CD49A (127, 130–133). CD8^+^ TRM cells are found in the entire reproductive tract and in contrast to CD8^+^ TRM cells in the kidney, skin and salivary gland, do not require IL15 for maintenance of the cell population (48, 49, 134).

Next to this, CD8^+^ TRM cells in the reproductive tract seem to be able to recruit circulating memory T cells, independently from their cognate antigen, into mucosal tissue of the reproductive tract and convert them to TRM cells (50). These data are suggestive of a well-functioning first line of defense of memory T cells in the reproductive tract. Presumably, TRM cells, CD4^+^ or CD8^+^, are the first memory T cells the male antigens on spermatozoa will encounter. Despite their presence in the reproductive tract, little information is available on their function and presence during pregnancy. One study looked at CD8^+^ TRM in endometrial tissue and showed the presence of high proportions of memory CD8^+^ cells in endometrial tissue, which was similar in women with recurrent miscarriages and control women (51). Part of these CD8^+^ memory T cells expressed CD103, indicating that the cells may be CD8^+^ TRM cells (51). The proportion of CD8^+^ memory cells expressing CD103 was similar in women with recurrent miscarriages and control women (51). However, the percentage of CD8^+^ memory cells expressing CD69, a TRM marker, was decreased in women with recurrent miscarriages as compared with control women (51). This might suggest a decrease in CD8^+^ TRM cells in women with recurrent miscarriage.

### CD8^+^ Regulatory Memory, CD8^+^ Follicular Helper Memory, and CD8^+^ Memory Stem Cells in Pregnancy

The regulatory memory, follicular helper memory, and the memory stem cell subsets are relatively well studied in the CD4^+^ cell compartment but only to a limited extent in the CD8^+^ cell compartment. CD8^+^ cells with immune regulatory abilities are described in literature (135–137), however, the existence of a memory cell subset within the CD8^+^ Treg cell population is still uncertain (45). CD8^+^ cells with expression of follicular helper cell marker CXCR5, and memory cell marker CD45RO, are identified in germinal centers of human tonsils, and were found to support B cells (116, 138). The presence of such CD8^+^ follicular helper memory cells are only very recently confirmed and are not studied in pregnancy yet (116). CD8^+^ memory stem cells, as for their CD4^+^ counterpart, are antigen specific memory cells with a naive phenotype and are mostly studied in oncology settings (115, 139–141). Research on CD8^+^ regulatory memory, CD8^+^ FHM, and CD8^+^ memory stem cells in pregnancy will be of interest, but more knowledge on their functioning in general is needed before studying their role in reproduction.

## Memory T Cells in Pregnancy as Possible Therapeutic Targets

Literature shows that memory T cells are likely implicated in fetal-maternal tolerance before, during and after pregnancy. Firstly, it has been shown that exposure to seminal fluid before pregnancy induces a memory T cell population in the ectocervix (122). Even though there is no evidence yet that these memory cells are paternal-antigen specific, this could be a mechanism that contributes to tolerance toward paternal-fetal antigens. This mechanism is supported by existing epidemiologic data showing an association between a longer period of exposure to seminal fluid of the future father and a lower risk of preeclampsia (142–144). Generating paternal specific memory T cells as a therapeutic target, through paternal cell immunization before conception seems obvious and has indeed been carried out by several studies (145, 146). Studies are however small, but a meta-analysis of 7 small studies showed an improvement in clinical pregnancy rate following IVF treatment when seminal plasma is used as an adjunct treatment (average pregnancy rate increased from 25% in the control group to 29% in the seminal plasma treated group), with no significant increases in live birth or ongoing pregnancy rate (146). Since in these studies, timed intercourse or deposition of untreated semen in the vagina before IVF was used, it is not known whether the positive effect of semen is due to the seminal plasma itself or to paternal-fetal antigen exposure. This should be subject of future research. In order to potentially achieve better results, additional options for priming may be tested. An additional option could be a prime and pull method, by first eliciting an immune response to recruit T cells into the reproductive tract, followed by topical vaccination, a method that has been shown to be effective in genital herpes prevention (147).

Secondly, this review indicates that tolerance mechanisms involving memory T cells are in place during pregnancy. Various alterations in memory T cell function and levels have been shown, which together likely ensure tolerance; for instance, CD4^+^ Treg memory cells may play an important role, while also low responsive PD-1^+^Tim-3^+^ CD8^+^ memory cells are present at the fetal-maternal interface, which may also be important. These different tolerating mechanisms and their interactions should be further investigated, while it is also important to focus on their alterations in complications of pregnancy. The lack of knowledge on these mechanisms in healthy pregnancy and how they are affected in complications of pregnancy, makes therapeutic options using immune modulation of memory T cells to treat pregnancy complications not feasible yet.

Thirdly, after pregnancy, maternal immune cells are exposed to fetal-paternal antigens through microchimerism and possibly through semen exposure (8, 9). Since CD4^+^ memory T cells are known to require low-levels of antigen exposure for long term maintenance (60, 148, 149), it is proposed that microchimerism and semen exposure are ways to ensure persistence of the fetal-paternal specific CD4^+^ memory cell population (150–152). Thorough investigations on possible beneficial effects of memory T cells on reproductive success and of microchimerism on memory T cell populations should point out whether this could bring forward another possible therapeutic target. Lowering pregnancy complication rates through priming and enhancing the maternal memory T cell repertoire in parous women could be considered for future therapies. These could involve similar approaches as therapeutic options before pregnancy.

## Conclusions

To conclude, a delicate balance of memory T cells seems necessary for successful pregnancy and memory T cells might not be harmful for pregnancy, but in fact, they may induce tolerance. Memory T cells show different phenotypes, dynamics, and functioning in uncomplicated pregnancies compared to memory T cells outside the reproductive context. Together, these mechanisms may induce tolerance toward fetal antigens during pregnancy. More research on memory T cells in pregnancy is needed to better understand the function of these cells in pregnancy and to develop therapeutic strategies for pregnancy complications based on memory T cells.

## Author Contributions

TK organized financial support, built the search strategy, performed the literature study, and wrote the first draft of the manuscript. AL organized financial support, built the search strategy, performed the literature study, and reviewed and edited the manuscript. SS, MF, and JP reviewed and edited the manuscript, organized financial support, and supervised the project.

### Conflict of Interest Statement

The authors declare that the research was conducted in the absence of any commercial or financial relationships that could be construed as a potential conflict of interest.
