# Supplementary material for: Memory T Cells in Pregnancy
Source: Front Immunol. 2019 Apr 2;10:625. doi: 10.3389/fimmu.2019.00625 (PMC6455355; doi:10.3389/fimmu.2019.00625)
Supplement: Supplementary file 1 [file Data_Sheet_1.PDF]

Supplementary Material

Search strategy

A systematic literature search was performed by searches on Pubmed, MEDLINE, Embase and the Cochrane database. Articles were selected from a list generated by combinations of the following search terms or their synonyms: reproduction, fetus, placenta, pregnancy, conception, fetal-maternal, insemination, sperm, memory, CD3, CD4, CD8, and T lymphocyte. Articles published in English between 1995 and 2019 were included.

With the search strategy 874 articles were found of which 860 were in English. 319 articles were selected based on the title. From these articles, all abstracts were read by two researchers (TK and AL) who selected 103 papers for full manuscript review. All 103 full manuscripts were read by both researchers and 40 were selected and mentioned in the review. Additional articles were included using the reference lists from the manuscripts found with the search strategy.
